# Popliteus Tendon Injury: A Rare Cause of Acute Locked Knee

**DOI:** 10.7759/cureus.38655

**Published:** 2023-05-07

**Authors:** Kin Chun Wong, Norlelawati Mohamad, Badrul Akmal Hisham Md Yusoff

**Affiliations:** 1 Department of Orthopaedics and Traumatology, Universiti Kebangsaan Malaysia Medical Centre, Kuala Lumpur, MYS

**Keywords:** anterior cruciate ligament, tendon injuries, popliteus tendon, knee injuries, locked knee

## Abstract

The acute locked knee is a common presentation of meniscal tears or other intra-articular injuries. However, a popliteus tendon tear, an uncommon cause of acute locked knee, is often overlooked as a possible diagnosis. Here, we present the case of a 29-year-old male who experienced an acute locked knee following a sports injury. An arthroscopic examination revealed an intrasubstance tear in the popliteus tendon and a complete anterior cruciate ligament tear, while the menisci remained intact. Due to the extension lag caused by the popliteus tendon tear, the anterior cruciate ligament reconstruction was postponed. The patient then underwent a course of physiotherapy before the anterior cruciate ligament reconstruction and eventually achieved full knee extension after six weeks. Further surgical intervention was then performed to address the ligament injury. Our case highlights the importance of considering a popliteus tendon tear as a possible cause of an acute locked knee. Proper diagnosis and management are crucial for achieving optimal outcomes for patients with an acute locked knee and concomitant ligamentous injury.

## Introduction

Suspecting an acute popliteus tendon injury can be challenging as it may present with subtle signs such as acute hemarthrosis without laxity and pain on the lateral aspect of the knee, or discomfort over the popliteus tendon [1-7]. Popliteus tendon injuries are increasingly recognized and often associated with concomitant ligament disruptions [8,9]. Missed injuries can increase the failure rates of both anterior and posterior cruciate ligament reconstructions, while untreated injuries may lead to chronic disability [6-8,10]. Locking of the knee due to popliteus tendon injuries is rarely reported in the literature. In this report, we present the case of a young male who suffered an acute locked knee due to a sports injury and was diagnosed with a popliteus tendon injury. The injury was treated conservatively, along with a concomitant anterior cruciate ligament (ACL) injury that was treated in stages. This case underscores the significance of achieving complete knee extension by addressing the underlying causes of acute locking and pseudo-locking of the knee before embarking on any ligamentous reconstruction surgery to ensure optimal outcomes.

## Case presentation

A 29-year-old male presented with an acute traumatic injury to his left knee sustained during a soccer game. The injury involved direct lateral trauma while the knee was flexed, followed by a twisting injury that caused an audible 'pop'. He experienced acute swelling and pain while weight-bearing and ambulating, resulting in an antalgic gait. His pain intensity was eight on the visual analog scale. Examination revealed an effused and tender left knee joint with loss of extension (loss of 30 degrees of extension), and the permissible range of motion was limited from 30 to 90 degrees. Further evaluation for ligamentous and meniscal pathologies was impeded by severe pain during manipulation.

**Figure 1:**
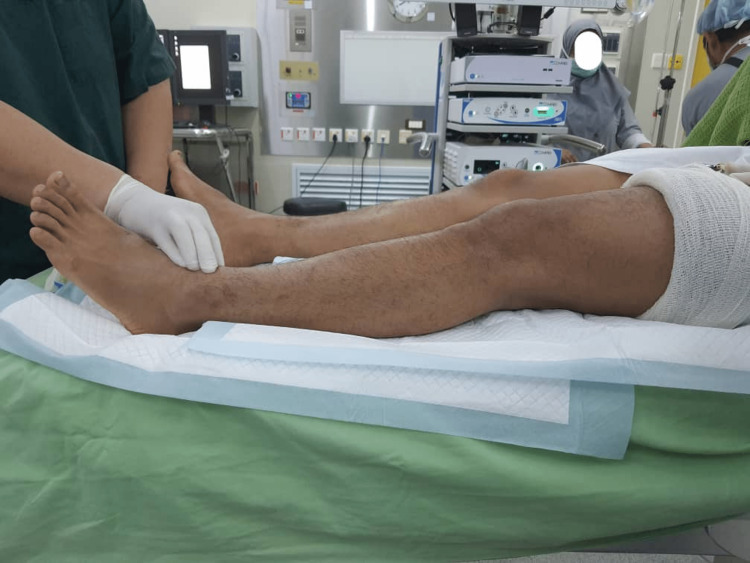
Spontaneous resolution of extension lag upon anesthesia.

**Figure 2:**
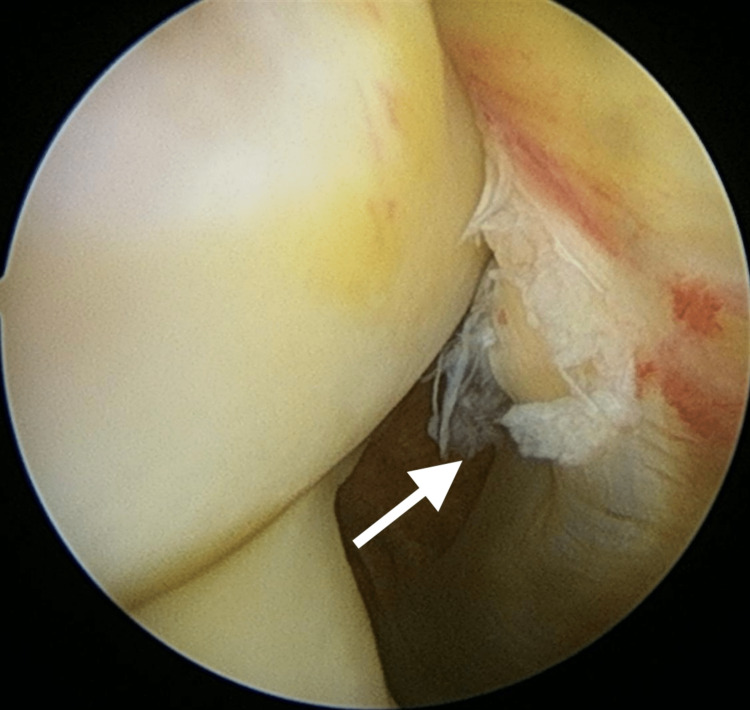
Arthroscopic findings of a partially torn popliteus tendon appeared inflamed. Arrow showing a torn popliteus tendon

**Figure 3:**
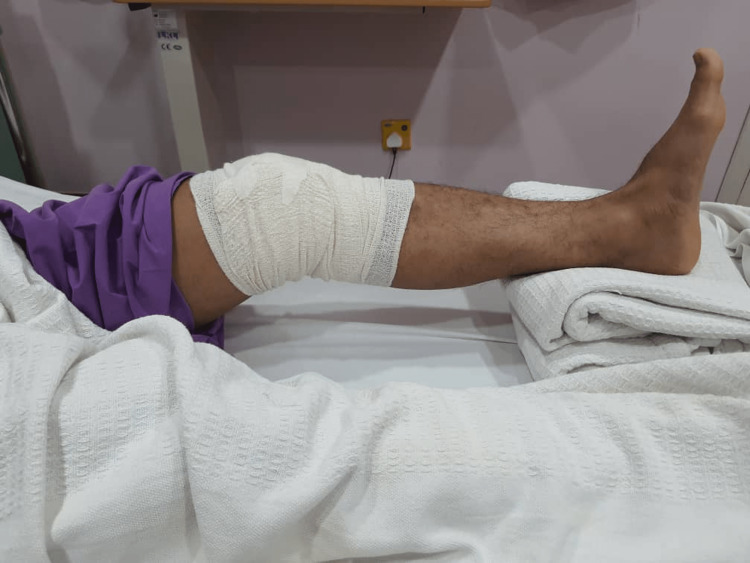
The knee resumed pseudo-locking with extension lag at 30 degrees upon regaining consciousness post-operatively.

As his knee was not favorable for positioning into the knee magnetic resonance imaging (MRI) coil, an arthroscopic examination was then performed on the sixth-day post-injury. Examination under anesthesia revealed resolution of extension loss (Figure 1), with a positive Drawer and Lachman's test indicating cruciate ligament instability. Other special tests, such as the varus test, valgus stress test, and McMurray test, were negative. Intraoperative findings confirmed a complete tear of the ACL, an intrasubstance tear of the popliteus tendon (Figure 2), and no meniscal or chondral injury. The synovium appeared healthy. Intraoperatively, full extension of the knee was achieved, but upon regaining consciousness, the knee spontaneously sprung back to 30 degrees of flexion (Figure 3).

**Figure 4:**
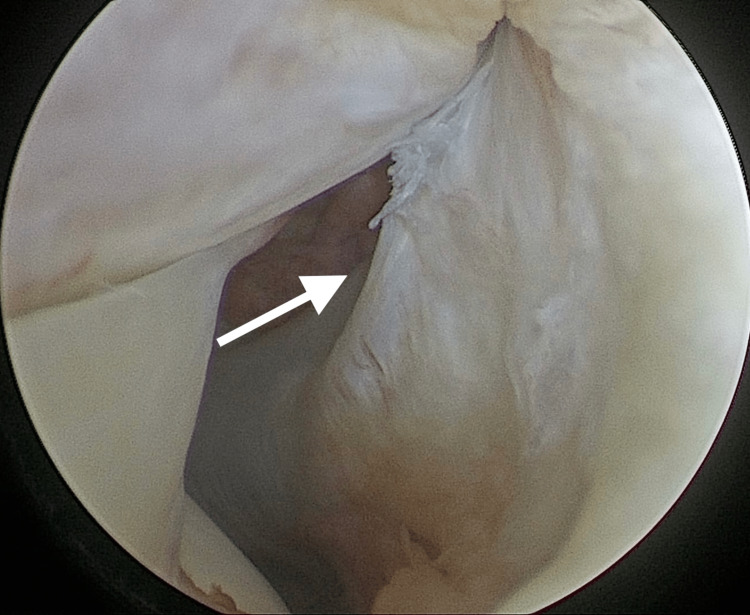
Arthroscopic findings of the scarred popliteus tendon. Arrow showing scarred popliteus tendon

The patient underwent a physiotherapy course aimed at reducing swelling and inflammation, increasing knee range of motion to full extension, and improving quadriceps strength. The prescribed therapy included 30 minutes of cryotherapy followed by 20-30 minutes of ankle pumps (10 repetitions per minute) with elevation. A set of exercises were included, such as passive range of motion stretches (supine knee extension, prone hangs, supine wall slides, seated knee flexion), isometric exercises (quadriceps and hamstring sets cocontraction), active-assistive range of motion (seated knee flexion), and active range of motion with progressive resistance exercises (heel raises, hip abduction, adduction, and external rotation). Gait training, with an emphasis on normal gait patterns and weight shifts, was gradually introduced throughout the physiotherapy sessions. After six weeks of physiotherapy, the patient regained full extension of the left knee and became pain-free. Examination showed no joint effusion or focal tenderness, except for the persistent laxity secondary to the ACL tear. ACL reconstruction was scheduled for two months later, once full extension of the knee was achieved. During the arthroscopic ACL reconstruction procedure, the popliteus tendon was re-examined and found to be scarred but otherwise intact (Figure 4). The ACL reconstruction and recovery were uneventful and successful.

## Discussion

The popliteus tendon is located intracapsularly but is extra-articular and extrasynovial. It is a part of the posterolateral corner of the knee and has biomechanical importance for knee stability [9-11]. The popliteus tendon complex comprises the popliteus muscle tendon unit and the ligamentous connections from the tendon to the proximal fibula (popliteofibular), the tibia (popliteotibial), and the meniscus (popliteomeniscal). The popliteal muscle originates from the posteromedial aspect of the proximal tibia and gives rise to its tendon, which courses intra-articularly through the popliteal hiatus of the coronary ligament to insert on the popliteal saddle on the lateral femoral condyle [6-8,10]. The tendinous portion of the popliteus traverses through the knee joint extrasynovially and is readily visible arthroscopically as it emerges from the popliteal hiatus [8,10,12].

Popliteus tendon injuries have not been commonly reported as a cause of extension loss. A locked knee can result from either an obstacle occupying the joint space, as seen in true locking, or hamstring contraction due to pseudo-locking [13-17]. Huri G and Bicer OS first reported a case of popliteus tendon injury presenting as traumatic acute locking of the knee. In the reported case, arthroscopic examination revealed that the popliteal tendon was partially injured, causing partial impingement in the popliteal hiatus postero-laterally. The authors managed the injury by debriding the ruptured tendon and gently removing the inflammatory tissue with a shaver. The patient reportedly regained an excellent outcome with a full range of motion and weight-bearing on the very next day after surgery [1]. Atraumatic causes of lock knee secondary to popliteus tendon rupture have also been reported, where an isolated avulsed tendon was identified arthroscopically and acted as a mechanical block. Arthroscopic excision of the loose tendon was proven effective in that particular case [13]. Another possible cause of a locked knee due to popliteus tendon pathology is acute calcific tendinitis, which is uncommon but reportedly responds well to local corticosteroid and anesthetic application [14].

The restricted range of motion in a locked knee can make it challenging to properly position the knee in the MRI coil and obtain clear MRI images of the joint. An arthroscopic examination is often preferred in these cases because it can provide both diagnostic and therapeutic benefits [12,18]. In our case, we performed arthroscopy to investigate the cause of the locked knee and address potential underlying issues, such as a meniscus bucket handle tear and an ACL tear with a tibial stump. Therefore, arthroscopy is a useful modality when MRI may not provide adequate diagnostic information or the patient's knee cannot be positioned into the knee MRI coil in the first place. However, if MRI is feasible, one should carefully look for the possibility of a partial popliteus tear [10,18].

Upon administering anesthesia, the knee was able to fully extend but sprung back to a flexed position upon regaining consciousness, indicating a pseudo-lock caused by a partially torn popliteus tendon. Our suspicion was further confirmed by ruling out any obstructions in the joint space during the arthroscopic examination. We opted for conservative treatment of the partially torn popliteus tendon and focused on regaining the full range of motion through physiotherapy to overcome the hamstring spasm. We will then address the torn ACL with ACL reconstruction once effusion subsides and the range of motion has been regained, typically a few weeks later. Generally, in cases where knee locking is caused by a popliteus tendon tear and there are no other structures or mechanical blockages, management includes allowing tendon healing for six weeks and relieving spasms. If an ACL tear is also detected, it would not be addressed during the acute phase due to the high risk of arthrofibrosis and suboptimal rehabilitation [19,20]. Proper assessment of the cause of locking remains a priority, and a high index of suspicion is crucial.

Our effective strategy for a pseudo-locked knee and complete ACL tear involved a thorough understanding and assessment of the patient's specific requirements and reconstruction prerequisites. By taking into account the potential complications of proceeding with ligament reconstruction without a well-structured plan, we were able to ensure a successful outcome. Once the patient overcame the pseudo-locking with the help of physiotherapy, we proceeded with the ACL reconstruction. During the re-examination of the popliteus tendon during ACL reconstruction, we found that the scarred tendon remained intact and was not inflamed. If we had proceeded with the ACL reconstruction without allowing the tendon to heal for a minimum of six weeks and failed to attain full extension, the recovery and rehabilitation would likely have been difficult and could have led to the failure of the ACL reconstruction. Overall, our approach emphasized careful consideration and precise execution to achieve optimal results for our patient.

## Conclusions

In summary, popliteal tendon injuries are potentially overlooked, and the treatment modalities vary according to the morphology of the injuries. Undiagnosed popliteus injuries may lead to poor rehabilitation after knee reconstructive surgery, which is avoidable. Popliteus tendon injury must be considered as a differential diagnosis and carefully managed if a locked knee is presented to clinicians.

## References

[1] Huri G, Biçer OS (2013). Unusual cause of knee locking. Case Rep Orthop.

[2] Conroy J, King D, Gibbon A (2004). Isolated rupture of the popliteus tendon in a professional soccer player. Knee.

[3] Westrich GH, Hannafin JA, Potter HG (1995). Isolated rupture and repair of the popliteus tendon. Arthroscopy.

[4] Radhakrishna M, Macdonald P, Davidson M, Hodgekinson R, Craton N (2004). Isolated popliteus injury in a professional football player. Clin J Sport Med.

[5] Guha AR, Gorgees KA, Walker DI (2003). Popliteus tendon rupture: a case report and review of the literature. Br J Sports Med.

[6] Ranawat A, Baker CL, 3rd 3rd, Henry S, Harner CD (2008). Posterolateral corner injury of the knee: evaluation and management. J Am Acad Orthop Surg.

[7] Zabrzyński J, Huri G, Yataganbaba A (2021). Current concepts on the morphology of popliteus tendon and its clinical implications. Folia Morphol (Warsz).

[8] Somanath D, Ramalingam S (2020). A comprehensive review of the anatomy of popliteus and Its clinico-surgical relevance. J Orthop Trauma Rehabilitation.

[9] Rosas HG (2016). Unraveling the posterolateral corner of the knee. Radiographics.

[10] Jadhav SP, More SR, Riascos RF, Lemos DF, Swischuk LE (2014). Comprehensive review of the anatomy, function, and imaging of the popliteus and associated pathologic conditions. Radiographics.

[11] Nyland J, Lachman N, Kocabey Y, Brosky J, Altun R, Caborn D (2005). Anatomy, function, and rehabilitation of the popliteus musculotendinous complex. J Orthop Sports Phys Ther.

[12] Mariani PP, Mauro CS, Margheritini F (2005). Arthroscopic diagnosis of the snapping popliteus tendon. Arthroscopy.

[13] Kheir E, Ghoz A, Gorgees K, MacDonald D, Limb D, Giannoudis P (2006). Spontaneous isolated rupture of popliteus tendon presenting as locked knee: case study and literature review. Eur J Orthop Surg Traumatol.

[14] Tibrewal SB (2002). Acute calcific tendinitis of the popliteus tendon--an unusual site and clinical syndrome. Ann R Coll Surg Engl.

[15] Allum RL, Jones JR (1986). The locked knee. Injury.

[16] Bansal P, Deehan DJ, Gregory RJ (2002). Diagnosing the acutely locked knee. Injury.

[17] LaPrade RF, Wozniczka JK, Stellmaker MP, Wijdicks CA (2010). Analysis of the static function of the popliteus tendon and evaluation of an anatomic reconstruction: the "fifth ligament" of the knee. Am J Sports Med.

[18] Zappia M, Reginelli A, Chianca V (2018). MRI of popliteo-meniscal fasciculi of the knee: a pictorial review. Acta Biomed.

[19] Rushdi I, Sharifudin S, Shukur A (2019). Arthrofibrosis following anterior cruciate ligament reconstruction. Malays Orthop J.

[20] Shelbourne KD, Wilckens JH, Mollabashy A, DeCarlo M (1991). Arthrofibrosis in acute anterior cruciate ligament reconstruction. The effect of timing of reconstruction and rehabilitation. Am J Sports Med.

